# Intestinal stem cells and gut microbiota therapeutics: hype or hope?

**DOI:** 10.3389/fmed.2023.1195374

**Published:** 2023-07-21

**Authors:** Ahmad Naqiuddin Ahmad Sophien, Amirah Syamimi Jusop, Gee Jun Tye, Yuen-Fen Tan, Wan Safwani Wan Kamarul Zaman, Fazlina Nordin

**Affiliations:** ^1^Centre for Tissue Engineering and Regenerative Medicine (CTERM), Faculty of Medicine, Universiti Kebangsaan Malaysia, Kuala Lumpur, Malaysia; ^2^Institute for Research in Molecular Medicine (INFORMM), Universiti Sains Malaysia, Gelugor, Malaysia; ^3^PPUKM-MAKNA Cancer Center, Universiti Kebangsaan Malaysia Medical Centre, Kuala Lumpur, Malaysia; ^4^M. Kandiah Faculty of Medicine and Health Sciences (MK FMHS), Universiti Tunku Abdul Rahman, Kajang, Malaysia; ^5^Department of Biomedical Engineering, Faculty of Engineering, Universiti Malaya, Kuala Lumpur, Malaysia; ^6^Centre for Innovation in Medical Engineering (CIME), Department of Biomedical Engineering, Faculty of Engineering, Universiti Malaya, Kuala Lumpur, Malaysia

**Keywords:** gut, microbiota, intestinal stem cells, microenvironment, regenerative medicine, mechanism

## Abstract

The vital role of the intestines as the main site for the digestion and absorption of nutrients for the body continues subconsciously throughout one’s lifetime, but underneath all the complex processes lie the intestinal stem cells and the gut microbiota that work together to maintain the intestinal epithelium. Intestinal stem cells (ISC) are multipotent stem cells from which all intestinal epithelial cells originate, and the gut microbiota refers to the abundant collection of various microorganisms that reside in the gastrointestinal tract. Both reside in the intestines and have many mechanisms and pathways in place with the ultimate goal of co-managing human gastrointestinal tract homeostasis. Based on the abundance of research that is focused on either of these two topics, this suggests that there are many methods by which both players affect one another. Therefore, this review aims to address the relationship between ISC and the gut microbiota in the context of regenerative medicine. Understanding the principles behind both aspects is therefore essential in further studies in the field of regenerative medicine by making use of the underlying designed mechanisms.

## 1. Introduction

Stem cells are a group of cells inside an organism that is yet to be differentiated into mature functional cells but have the capabilities to differentiate into any kind of cells, alongside being self-renewal (1). These abilities in differentiation into any kind of cells and self-renewal of stem cells prove to be important in developing and regenerating certain cells in the body. Stem cells are grouped by highest differentiation ability, in descending order: totipotent, pluripotent, multipotent, oligopotent and, lastly, unipotent (2), which is from being able to generate an entire organism and extraembryonic tissues, to only a single lineage of cells.

Totipotent cells are stem cells that can differentiate into all types of cells in an organism, together with the development of extraembryonic tissues such as the placenta (3). Pluripotent cells are stem cells that can differentiate into any three distinct germ layers: endoderm, mesoderm, or ectoderm (4). Each layer is responsible for specific organogenesis, such as the endoderm layer that develops the gastrointestinal tract, liver, pancreas, the mesoderm layer into the circulatory system and the musculoskeletal system, and the ectoderm responsible for hair, skin, nails, and nervous system (5). Multipotent stem cells are also known as adult stem cells or somatic stem cells. Examples of multipotent cells in the human body include but are not limited to, hematopoietic stem cells, mesenchymal stem cells, skeletal stem cells, neural stem cells (6), and intestinal stem cells (7). Similarly, oligopotent and unipotent stem cells also contribute to their tissue-specific cell lineages, capable of differentiation into a limited number of cells or terminally differentiated (1).

Continuing from the multipotent stem cells, examples of stem cells with this differentiation ability are intestinal stem cells (ISC), which are undifferentiated stem cells located in the crypts of intestinal epithelium (8). These stem cells are the cells that give rise to the intestinal epithelium with its variety of cells and many more functionalities by continuously proliferating and dividing (9). From the ISCs, the progenitor cells continue to divide and differentiate into specific cell lineages such as absorptive or secretory cells (10). Facilitation of these processes is crucial to balance both the proliferation of new cells and the removal of old or damaged cells, which is provided by the plethora of appropriate biochemical signaling pathways. Without it, DNA mutations can especially accumulate in cells, leading to uncontrolled differentiation and potentially producing harmful cancerous cells (11).

The gut microbiota refers to a dynamic community of microorganisms that live in the human gastrointestinal tract (12). These consist of various bacterial species, viruses, archaea, and fungi. As the gastrointestinal tract, particularly the stomach, small intestine, and large intestine, are responsible for the digestion and absorption of food and drinks ingested, this provides a perfect environment for microorganisms to colonize the site of the nutrients and safety provided by the human host (13, 14). Despite the enormous number of microorganisms in the gastrointestinal tract (GI), a large proportion of them have a symbiotic relationship with the human host through many biological processes. For example, the gut microbiota helps mediate body homeostasis, strengthen the integrity of the gastrointestinal tract, and decrease the colonization of foreign or pathogenic strains of microorganisms in the GI tract (15). Furthermore, the gut microbiota regulates the innate and adaptive components of the immune system such as antigen-presenting cells, regulatory T cells, and innate lymphoid cells (16).

Unlike any other stem cells, intestinal stem cells exist in tandem with the indigenous gut microbiota population in the GI tract, albeit living separately within their integrity and maturation of the intestinal epithelium depend on the harmonization of both the intestinal stem cells and the gut microbiota working together. The absence of either will disrupt the equilibrium between the two, either with dysbiosis, the imbalance of the gut microbiota, or inflammatory bowel disease (17).

**Figure 1:**
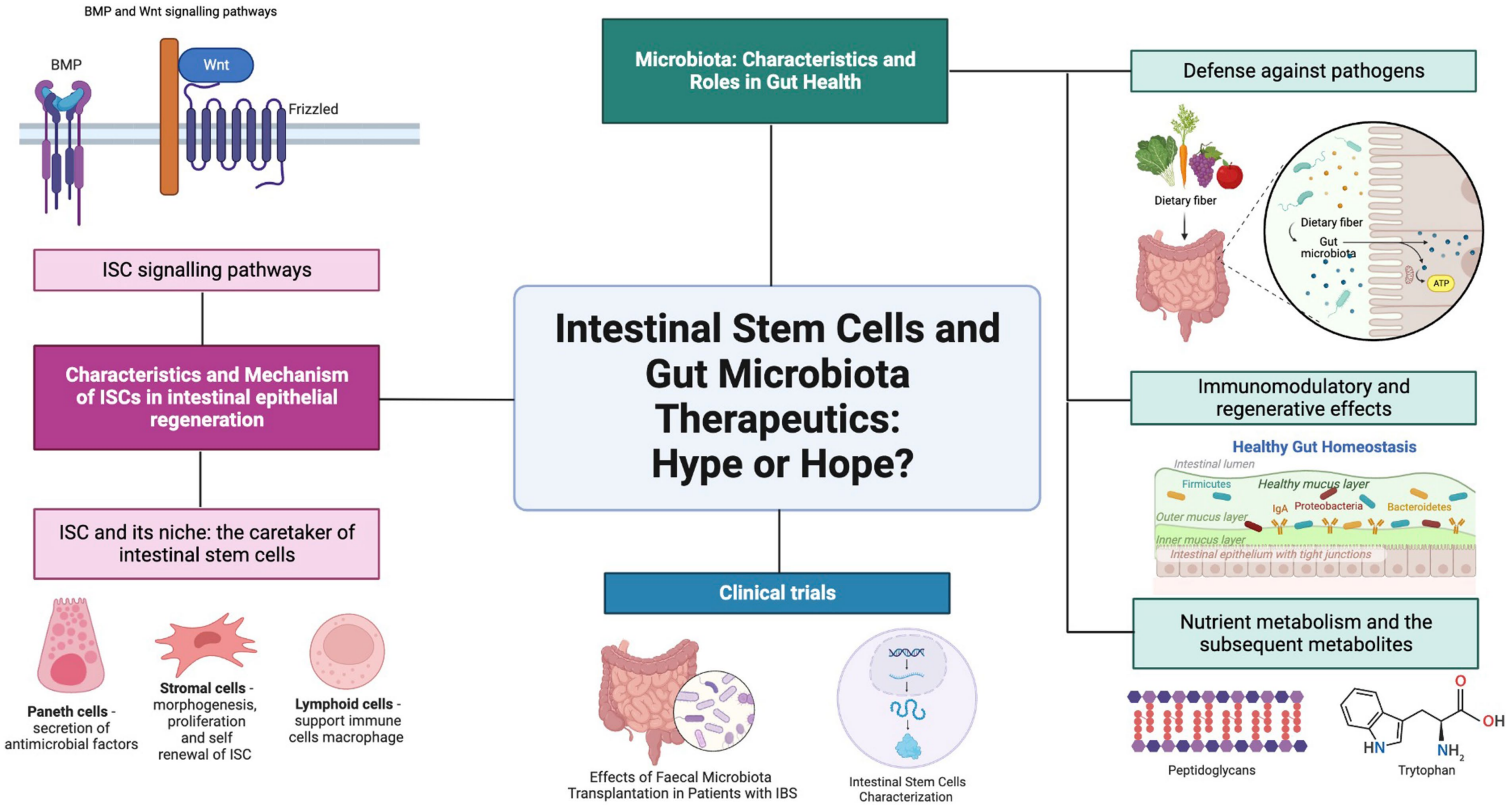
Graphical abstract. Gut Microbiota and ISC. Created with BioRender.com.

Hence, this review aims to address the relationship between ISC and gut microbiota within the context of regenerative medicine. The characteristics of ISC are explained thoroughly by investigating the Lgr5 and + 4 stem multipotent intestinal stem cells that all intestinal epithelial cells are derived from. The diverse bacterial and fungal community that makes up the gut microbiota in the human gastrointestinal tract is also investigated for the roles they play in the host organism. The relationship between the ISC and the gut microbiota is then further examined to determine their combined roles in regulating the homeostatic function of the intestine. A clinical trial is also included on ISC and gut microbiota to highlight these specific research studies as summarized in (Figure 1). Future perspectives and recommendations are also discussed on progress in the field of study.

## 2. Characteristics and mechanism of ISCs in intestinal epithelial regeneration

The intestines have been able to perform their function of digestion and absorption of nutrients for the body due to the meticulous architecture of various cells, substances, and their microenvironment. Both the small and the large intestine are composed of a single layer of columnar cells that line the epithelium of both organs. The small intestine also has villi protruding into the lumen and Lieberkühn crypts, while the colon also has the crypts and absorptive plates which are alternatives to villi, with these features to increase the total surface area of cells that touch the food ingested (18). These crypts house intestinal stem cells (ISCs), which all the intestinal epithelial cells originate from (9).

Intestinal epithelial cells, also known as mature intestinal cells, consist of two lineages: the absorptive lineage and the secretory lineage (10). The absorptive lineage with enterocytes, whose main function is to absorb nutrients from food, and M cells or microfold cells are to continuously examine intestinal microbes and mediate the appropriate mucosal immune response (8, 19). Secretory lineage cells comprise four types (i) enteroendocrine for hormone production and release, (ii) Goblet cells for mucus production and secretion, (iii) opioid-secreting Tuft cells (20) and (iv) multifunctional Paneth cells for the secretion of antimicrobial peptides as well as signaling molecules to facilitate the maturation of intestinal stem cells and their subsequent progenitor cells (9, 21).

As mentioned above, mature epithelial cells are derived from multipotent intestinal stem cells that reside in the crypt of the intestinal epithelium. The turnover of intestinal epithelial cells is maintained by differentiation of ISCs into different cell lines of secretory or absorption by maturing first into the respective progenitor cells (22, 23); also known as transit amplifying cells (TA cells). These secretory or absorptive progenitor cells can be further committed to their respective lineage and differentiate into secretory Paneth cells or absorptive enterocytes, respectively (24). ISCs, TA cells, and other types of absorptive and secretory cells are located in the single layer of the intestinal epithelium. All except ISCs and Paneth cells continuously move across the crypts and villi and toward the lumen before ultimately shedding into the lumen (25).

**Figure 2:**
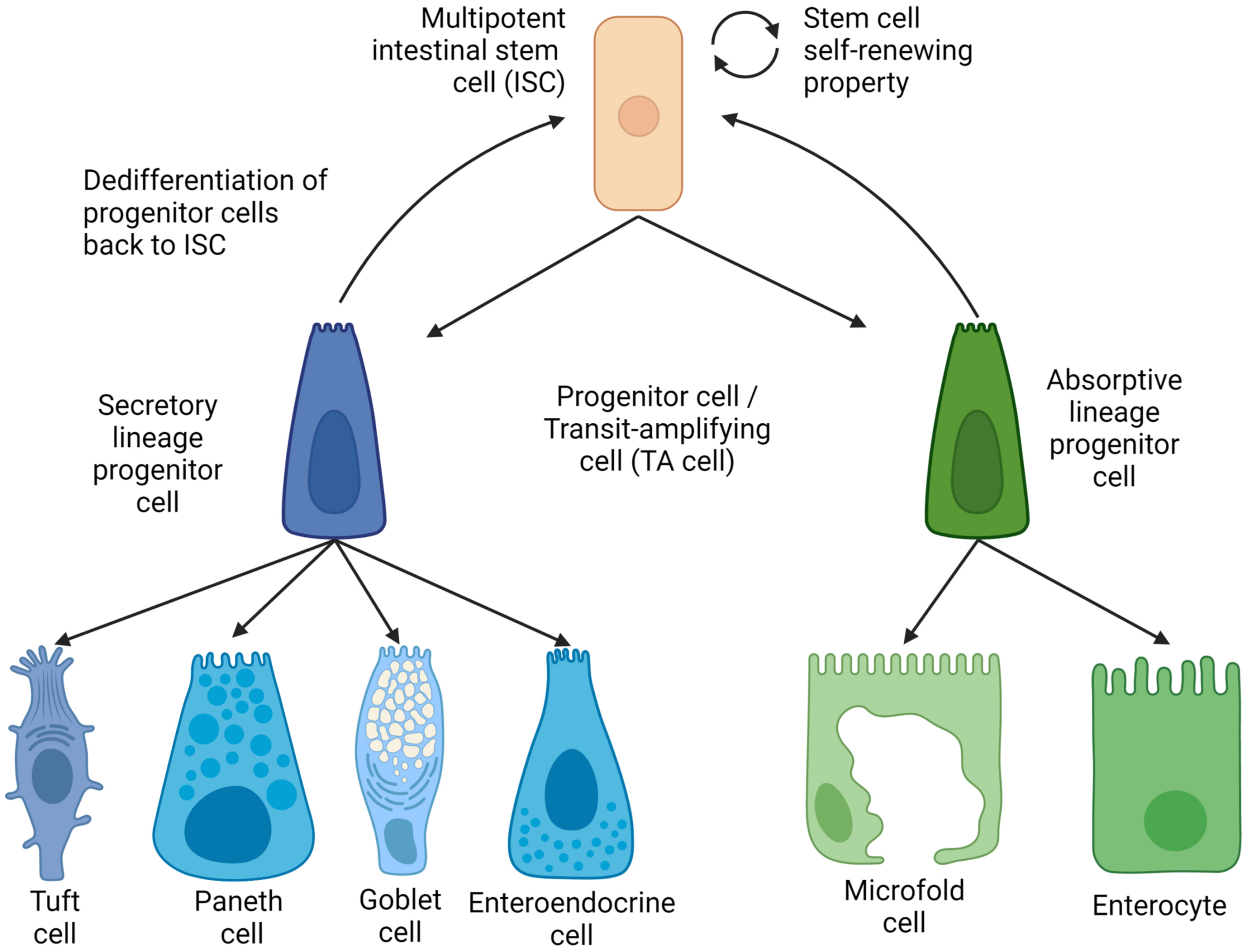
Intestinal stem cell lineages and differentiation. Created with BioRender.com. The figure is adapted with modification from Haoming Luo et al. (9).

The intestinal stem cells were first discovered to be located in between Paneth cells at the bottom of a mouse intestinal crypt in alternating patterns. These cells were coined as crypt-base columnar cells (CBCs) and demonstrated their stemness, which is the ability of the cells to proliferate and differentiate. This can be confirmed by the fact of a similar radioactive component that was observed in intestinal cells that differentiated from the CBCs (26). The gene Lgr5+ was discovered as one of the genes specific to CBCs (27) during the investigation of the Wnt signaling pathway as an important homeostatic pathway in intestinal regeneration. These LGR5-expressing cells were collected from intestinal crypts and continuously self-renew and were also capable of differentiating into the two main intestinal cell lines and are long-lived under conditions enhanced by specific growth factors (20, 28, 29). Thus, the self-renewal and the ability to differentiate into multiple lineage-specific progenitor cells meet the criteria of stemness as described in (Figure 2).

Besides Lgr5+ stem cells, another distinct type of intestinal stem cell called +4 stem cells are found in the ISC. The name of the +4 stem cells derives from the position of this cell type, as they are the ‘fourth’ from the bottom of the crypt (30). These +4 stem cells are observed to be more quiescent than Lgr5 ISCs and were generally regarded reserve stem cells that help replenish the actively used Lgr5 stem (31), either by generating intestinal progenitor cells (32) or by dedifferentiating transit-amplifying cells back into Lgr5 stem cells with the transcription factor ASCL (33). It has been observed that the Lgr5 stem cell pool can be replenished by both absorptive and secretory progenitor cells, and mature Paneth cells were also found to be capable of de-differentiating (34–36).

### 2.1. Intestinal stem cells signaling pathways

Many factors and niches are involved in regulating the action of the intestinal epithelial cells, their response to injury, as well as the subsequent corresponding actions from the nearby ISCs (37). At the intracellular level, the signaling pathways that are important for the homeostatic function of ISCs are the Wnt, BMP, and Notch signaling pathways (38).

**Figure 3:**
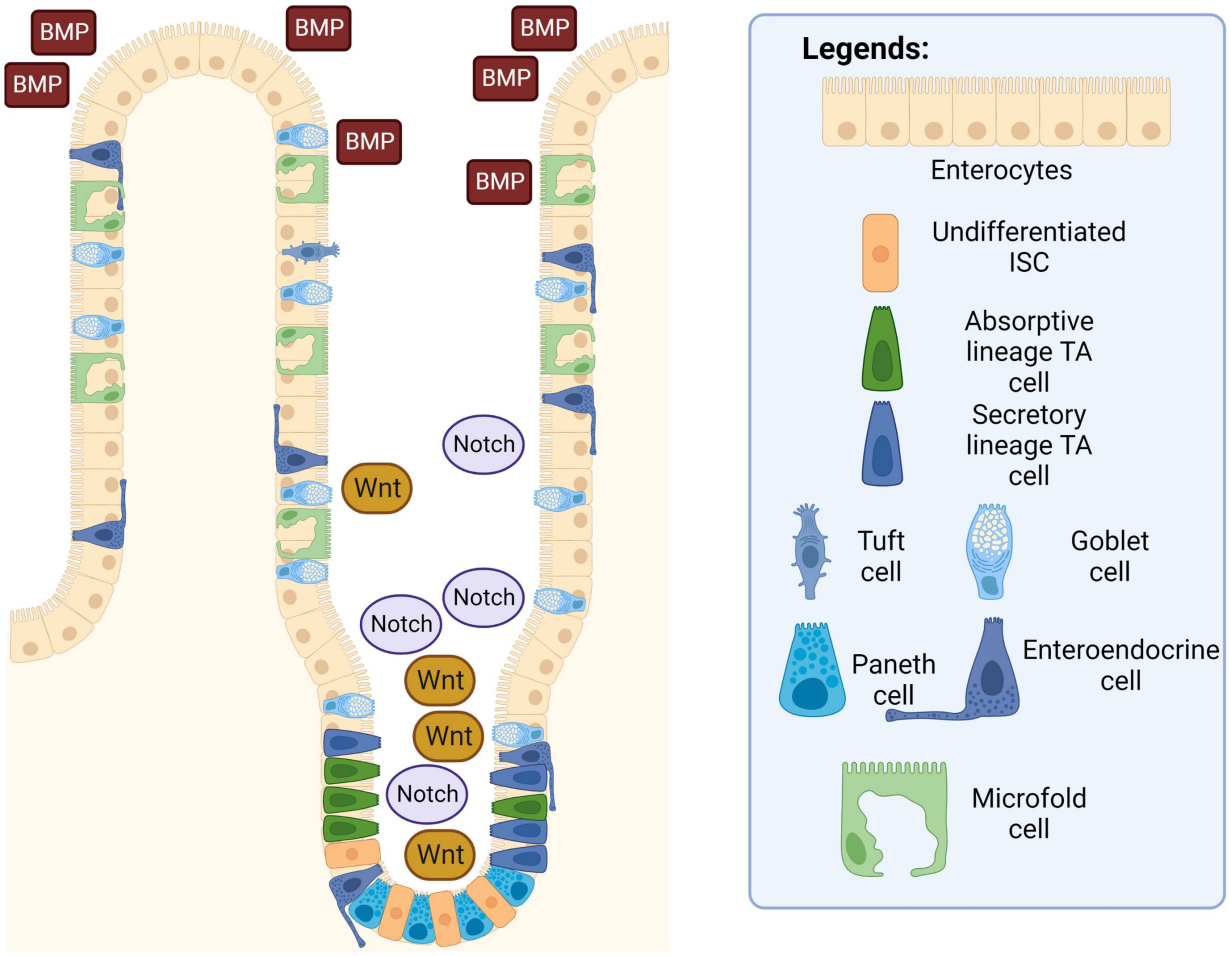
Intestinal epithelium with ISC signaling pathways. The Wnt and Notch signaling pathways are in a higher concentration in the intestinal crypts, while the BMP pathway is more concentrated near the villus. Created with BioRender.com. The figure is adapted with modification from Haoming Luo et al. (9).

The Wnt signaling pathway in (Figure 3) is found to be responsible for the promotion of ISC self-renewal and proliferation in intestinal crypts, which decreases dramatically once cells reach the villus (39). This pathway is intricately modulated by the degradation of the β-catenin protein by the β-catenin destruction complex, consisting of multiple proteins such as AXIN, casein kinase 1 (CK1), glycogen synthase kinase 3, and adenomatous polyposis coli (APC). Studies have shown that mutations in adenomatous polyposis coli (APC) destruction complexes along this pathway are correlated in multiple cases of colorectal cancers (40). APC is a tumor suppressor gene that is essential in the regulation of the Wnt signaling pathway; and irregular and mutated APCs are commonly observed in colorectal cancers (41). When there is a mutation in the structure of the APC protein, another protein that is usually inhibited by the APC protein complex called USP7 allows the deubiquitination of the β-catenin, thereby leading to excessive growth and proliferation of ISCs that can develop into cancerous tissues. Additionally, Wnt signaling resume despite with the removal of the upstream USP7 gene by CRISPR, thus showing a therapeutic potential in administering functional gene and their derivative proteins to the intestinal tratcs to possibly cure participants with gastrointestinal cancer and once approved, to the public as well.

The bone-morphogenetic protein (BMP) signaling pathway tightly regulates the proliferation of ISCs to prevent out-of-control cell differentiation, crypt fission, and handling the terminal differentiation of mature and functional intestinal cells. Signaling was also observed to be higher around terminally differentiated cells in the villus to prevent the uncontrolled dedifferentiation of these aged cells back into progenitor cells. In other words, epithelial cells in the gastrointestinal tract are unique compared to other tissues, since epithelial cells are capable of dedifferentiate and subsequently acquire stem cell-like properties and differentiate into other specific intestinal progenitor cells through actions in the BMP signaling pathway (42). Additionally, a suppressed or inhibited BMP signaling pathway has been shown to cause the appearance of abnormal intestinal crypts and villi in mice, thereby demonstrating that the BMP pathway is essential in decreasing hyperplasia of intestinal epithelium (43, 44).

On the other hand, the Notch signaling pathway works together with the Wnt signaling pathway to promote ISC proliferation and intestinal epithelial regeneration. When there is an injury to the intestinal epithelium, the signaling pathway is activated to promote the repair and regeneration of the epithelial surface via the proliferation of stem cells (45). A similar study has also shown that an inhibited Notch signaling pathway in mice correlates with an increase in apoptotic cell rates and fewer columnar cells in intestinal crypts. The inhibited pathway leads to a decrease in intestinal functionality of mice, as several of them experienced an excessive reduction in body weight, with even two mice dead before scheduled euthanasia, which can be attributed to the lack of intestinal epithelial regeneration due to inhibition of the Notch signaling pathway (45). Furthermore, the Notch signaling pathway also plays a decisive role in lineage differentiation (46). ISCs can be influenced to differentiate into the secretory lineage by expression of certain genes, such as the hairy and split enhancer [Hes1] (47), which in turn repress the transcription factor mouse atonal homolog 1 [Math1] (48). Similarly, stem cells can differentiate into an absorptive lineage when the two genes are removed (49). In short, Wnt and Notch signaling pathways promote the proliferation of ISCs into intestinal progenitor cells, and BMP signaling pathway acts as the regulator to prevent over-differentiation and hyperplase of these intestinal epithelial cells. Additionally, Notch signaling pathway also serve as a guiding system of which intestinal lineage the ISC should differentiate into.

### 2.2. Intestinal stem cells and its niche: the caretaker of intestinal stem cells

In addition to the three main regulatory pathways that are responsible for the homeostatic functions of the ISCs, these stem cells are also regulated and taken care of by the surrounding niche cells in the crypts, namely stromal cells, endothelial cells, Paneth cells, neural cells and immune cells. These groups of cells, collectively known as the stem cells niche, are believed to be responsible for maintaining the number of stem cells and their fate through terminal differentiation into absorptive or secretory lineages, and self-renewal (50).

Paneth cells are cells located interdigitally between the Lgr5 intestinal stem cells of Lgr5 in intestinal epithelial crypts and are important cells in the niche, as these cells are believed to maintain a sterile environment for stem cells through the secretion of antimicrobial factors (9). Homeostasis of the intestinal epithelium is believed to be maintained by Paneth cells is by sensing bacterial metabolites from the indigenous microbial community such as lactic acid on the protein receptors of the Paneth cells, which can activate the release of Wnt3 into the intestinal lumen. Subsequently, epithelial regeneration and ISC proliferation are initiated through the Wnt signaling pathway, ensuring that intestinal functionality in absorbing nutrients continues (51). Furthermore, when prostaglandins are produced in an autocrine signaling pathway from the Paneth cells, the Wnt pathway is also stimulated to promote the self-renewal and proliferation of ISCs in committed progenitor cells (52). Surprisingly, the expression in this regulatory pathway is believed to be connected to the exposure of certain bacterial components such as bacterial lipopolysaccharides and IL-6 bacterial immune cytokines (53–55), thus suggesting that Paneth cells secrete regulatory factors when certain bacterial components are detected in the lumen, further solidifying the important role that Paneth cells have in the homeostatic function of ISCs.

Stromal cells are a group of connective tissues that are of non-epithelial and non-endothelial origin but still play a role in helping organ function. Studies have suggested that stromal cells such as myofibroblasts that reside beneath the intestinal crypts known as lamina propria are involved in the morphogenesis, proliferation, and subsequently, differentiation and self-renewal of ISCs through various methods (56, 57). Researchers from Hubrecht Institute for Developmental Biology and Stem Cell Research have argued that the development of intestinal epithelium, simulated using intestinal organoids, is possible either through coculture with stromal cells or the addition of external Wnt (58). This is further supported by another study that discovered the expression of an agonist of the Wnt signaling pathway from endogenous stromal cells, possibly highlighting the mechanism by which stromal cells support the regeneration of the intestinal epithelium even without Wnt secreted by the epithelial layer (59). Moreover, in another study in which the subpopulation of mesenchymal cells called telocytes in mice is experimentally depleted through the administration of diphtheria toxin, the depth of the crypt and the height of the villus, along with the rate of stem cell proliferation, have been observed to be reduced at the end of the study (60), underlining the important role and relationship that stromal cells have with the intestinal epithelium.

Innate lymphoid cells represent an important population of the stem cell niche. Living in the lamina propria layer, one particular subtype of ILC called ILC3s has been extensively researched on to produce cytokine IL-22 from the simulation of the gut microbiota with different responses depending on the target cells on the intestinal epithelium (61). When IL-22 is treated on TA cells, differentiation, and proliferation into committed progenitor lines were observed, but on ISCs, decreased survival rates and reduced Notch and Wnt signaling was observed instead (62, 63). Therefore, IL-22 is believed to be essential for the survival of ISCs. However, the process by which ILC3 is activated to produce protective cytokines through signals from the gut microbiota requires further investigation. Additionally, T-lymphocytes play an important role in keeping the microenvironment of the intestinal lumen sterile, particularly at the crypt housing the ISCs and the progenitor cells. T cells reside in the lamina propria of the intestines and are dual-controlled by the human host antigens and indirectly by the gut microbiota. When there is an external pathogenic infection, Th1 and Th2 cells support immune cells macrophage to kill vesicular bacteria and help B cells in the activation and antibody production, respectively. In the process, cytokines are also released to induce differentiation of ISC to Tuft and Paneth cells to further locate and eliminate pathogens, while simultaneously inhibiting their own stem cell proliferation. In the same study, the researchers have also advocated that T cells may also communicate via MHC class II molecules with ISC which results in the production of the cytokine IL-10 that encourages self-renewal of ISC (64).

## 3. Microbiota: characteristics and roles in gut health

The gut microbiota refers to the abundant and complex collection of complex microorganisms that reside in the gastrointestinal tract. These consist of a diverse group of bacteria, viruses, archaea, and fungi, which can reach a staggering number of 100 trillion microbes when accumulated (14). As the GI tract, particularly the stomach, small intestine, and large intestine, are responsible for the digestion and absorption of ingested food and drinks, this provides a perfect environment for microorganisms to colonize the site for the nutrients and safety provided by the host (13, 14).

It is important to note that the gut microbial community is enormous and diverse; therefore, a single microbiota composition is not properly optimized, as it is unique individually. These depend on many factors such as early life factors such as type of delivery, milk feeding from the mother and weaning period, as well as external factors such as frequency of exercise, sociocultural diet habits, BMI level, and enterotypes (12).

Besides, variations in the gut microbiota intra individually can also be affected such as in certain anatomical regions. For example, the short transit time of the intestine of 3 to 5 h and the high concentrations of bile in the small intestine prove to be a difficult environment for microbes to thrive. Thereby, a study has revealed that facultative anaerobes such as gram-positive *lactobacilli*, *enterococci*, and *streptococci*, as well as gram-negative *Bacteroides* and *Proteobacteria*, were observed through molecular analysis to populate the jejunum and ileum region (65, 66). On the contrary, the neutral to mildly acidic pH and the slow flow rate of the large intestine harbors a more diverse gut microbiota, mainly consisting of obligate anaerobes. In healthy individuals, there is minimal microorganism colonization on the surfaces of the large intestinal epithelium and an inner mucin layer. The distal mucin layer has bacteria with special mucin-degrading properties, such as *Akkermansia muciniphila* and *Bacteroides* spp., which are part of the normal microbial flora, the former having immunomodulatory responses in mouse models (67, 68).

Primarily, small metabolites that are produced from their metabolism are the main essential method in which the gut microbiota can indirectly influence the intestinal epithelial layer function and development. Direct exposure of ISC to gut microbes, indigenous or not, can be devastating considering the vital role of ISC and progenitor cells’ long-term integrity and functionality. Therefore, the main method of the effect of the gut microbiota on ISC is mediated by their interactions with intestinal epithelial cells in the upper epithelium layer, which will subsequently cause the appropriate cascade of responses in various feedback systems that can protect ISC and progenitor cells from biological damage or promote regeneration of the epithelial layer through differentiation and proliferation. Despite the enormous number of microorganisms in the gastrointestinal tract, a large proportion of them have a symbiotic relationship with the human host through many biological processes (15). This includes, but is not limited to, the defense against pathogenic microbes, the immunomodulatory effects of sending signals to host immune cells and helping in host nutrient metabolism (69).

### 3.1. Defense against pathogens

Gut microbiotas offer the host a pathogen resistance against infectious bacteria as both commensal gut microbiota and pathogenic bacteria must compete for the same limited nutrient, habitat, and energy that is in the host gastrointestinal tract to grow. For that, the indigenous gut microbiota has been found to evolve and use certain methods to restrict the growth of foreign microbes through direct and indirect pathways (70). Direct pathway refers to the indigenous intestinal microbes being directly responsible for the protection of the GI tract against pathogenic bacteria growth and colonization by altering the pH of the GI tract (71–73), producing harmful chemical substances that are toxic to the pathogens (74, 75), and competing for the limited available nutrients (76, 77). This is done through the secretion of specific bacterial metabolites that favor the growth of the indigenous gut flora while discouraging the growth of potentially harmful strains and species. For the indirect mechanism, the gut microbiota is believed to acquire the help of the host immune system to eliminate foreign pathogens and bile acid metabolism that mediate the maintenance of the intestinal epithelium layer (78, 79) through recognition and the response to pathogen recognition receptors (PRRs) (80). When the healthy microbial community is disrupted either through antibiotics (81), age, dietary patterns, or from the host’s genetics (82, 83), this can cause an imbalance in the delicate arrangement of the gut microbiota also known as dysbiosis, thus it may lead to unhealthy outcomes such as inflammatory bowel disease (IBD), infections from opportunistic pathogens, allergies, obesity, and cardiovascular disease (84, 85).

### 3.2. Immunomodulatory and regenerative effects

It is important that the physiological system of the human host can distinguish between the beneficial or commensal gut microbiota of the indigenous population with pathogenic or opportunistic microbes. An abnormal immune response such as chronic inflammation can therefore be avoided or minimized by maturing a properly functioning immune system. This includes both the general and specialized immune responses, where the former is responsible for the epithelial physical barrier and producing chemicals that circulate continuously to identify and destroy a wide variety of potentially pathogenic organisms or their antigens (86). These have been studied extensively through the use of germ-free animal models (87) and animals treated with large concentrations of antibiotics that essentially reduce the number of indigenous gut microbes (88, 89).

In contrast, infection from toxic and harmful bacterial strains can impede the intestinal epithelium regeneration, such as infection from the diarrhea and colitis-causing *Clostridium difficile* can injure the intestinal epithelium and with the toxic metabolite TcdB (90). These have been studied extensively through the use of germ-free animal models (87) and animals treated with large concentrations of antibiotics that essentially reduce the number of indigenous gut microbes (88, 89). In contrast, Wang et al. have discovered that the “good-bacteria” *Lactobacillus reuteri* D8 can stimulate the recovery and growth of the intestines and gut organoids after experimentally damaging them with TNF-α. This positive effect was observed through the increase in Lgr5, Ascl2, and Olfm4 from the Lgr5+ ISCs after exposure to the good bacteria *L. reuteri*, which resulted in a greater number of Paneth cells and the regeneration rate of Lgr5 ISC. Further studies also revealed that the probiotic *L. reuteri* also can stimulate the Wnt/β-catenin signaling pathway contributing to further stimulation of the epithelial proliferation via Lgr5 ISCs (91). Additionally, erythroid differentiation regulator-1 [Erdr1] is an important protein that plays many roles in cell growth homeostasis such as cell proliferation, regulating cell apoptosis, and regeneration rate (92). Expression of this protein is only achieved with the help of the gut microbiota that inhabits the intestine during the early years of life, which in particular helps regulate the role of the protein expression in the proliferation of Lgr5 ISCs (93). After a considerable amount of time, researchers were finally able to culture *in vitro* segmented filamentous bacteria that is critical for immune development (94, 95). Such studies demonstrate the importance of the gut microbiota in maturing and thus indirectly regulating the host immune response in the intestines.

### 3.3. Nutrient metabolism and the subsequent metabolites

The plethora of bacterial phyla such as *Bacteroides*, *Actinobacteria*, *Firmicutes*, and *Proteobacteria* shown in Tables 1–3 comprises the majority of indigenous gut microbiota populations in the intestinal tract (96), and these microbes require nutrients in order to stay alive and perform their metabolic actions. These microbes rely on undigested dietary substances or unabsorbed nutrients such as carbohydrates (97), proteins, peptides, and dietary fibers as their energy source (98). From these metabolisms, metabolites are produced subsequently, and it is from these metabolites that can interact with the epithelial cells, particularly of ISC via indirect modulatory processes. Examples of such metabolites are short-chain fatty acids, tryptophan metabolites, and peptidoglycans (99).

**Table 1:** Enterotypes of intestinal bacteria in the GI tract.

Phyla	Genus	References
*Firmicutes*	*Lactobacillus*	(126)
	*Bacillus*	
	*Clostridium*	
	*Enterococcus*	
	*Ruminococcus*	
*Bacteroidetes*	*Bacteroides*	(126)
	*Actinobacteria*	
	*Proteobacteria*	
	*Fusobacteria*	

**Table 2:** Fungus enterotypes of the gut microbiota in the GI tract.

Genus	Species	References
*Candida*	*C. albicans*	(127)
	*C. tropicalis*	(128)
	*C. parapsilosis*	
	*C. glabrata*	
“Colonizers” [not indigenous to GI tract]	*Malassezia [genus]*	(128)
	*Cladosporium*	
	*Aspergillus*	
	*Penicillium*	

**Table 3:** Archea enterotypes of gut microbiota in GI tract.

Archaea	Family (in order of most abundant)	References
	*Methanobacteriaceae*	(129)
	*Haloferacaceae*	
	*Methanomethylophilaceae*	
	*Euryarchaeota*	

Plant fibers can be fermented by *Firmicutes* and *Bacteroidetes* to produce ethanol, lactate, hydrogen, carbon dioxide, and most importantly, short-chain fatty acids (SCFAs) (100). Studies have shown the importance of SCFAs in the homeostatic physiological function of the human body (101) such as butyrate being the energy source of epithelial cells in the colon (102), signaling to receptors involved in the regulation of appetite control hormones such as glucagon peptide 1 (GLP1) and peptide YY (PYY) (103), and having potential anticancer effects by stimulating apoptosis of cancerous colonic cells (104). Furthermore, SCFA has also been studied in detail for its ability to indirectly affect ISCs by limiting the proliferation rate of the progenitor cells to outcompete or block access to the epithelium’s critical receptors/binding sites (105, 106).

Tryptophan from dietary intake such as milk (107) plays an important role in the indirect production of aryl hydrocarbon receptors [AhR] in intestinal epithelial cells. The gut microbiota (108), such as *Acinetobacter oleivorans* (109), *Vibrio cholerae*, *Lactobacillus* spp., and *Chromobacterium violaceum* (110, 111) are mainly responsible for the metabolism of tryptophan to generate indoles and their derivatives. These derivatives could stimulate the production of AhR receptors which in turn are important for maintaining normal reflux in the intestines and preventing the uncontrolled proliferation of ISCs that can lead to tumors (112). A study in elderly mice with an irregular AhR pathway revealed abnormal and excessive proliferation of ISCs that can risk the generation of tumorigenic cells, due to the pathway interfering with the main Wnt/β-catenin signaling (112).

Peptidoglycans which constitute the bacterial cell wall of the gut microbiota are involved as immunomodulators and the maturation of the intestinal epithelium. Muramyl dipeptide [MDP], which is an active component in the peptidoglycan, may prevent the programmed apoptosis of Lgr5+ ISCs from oxidative stress and simultaneously induce the Lgr5 ISCs proliferation. During injury, MRP is found to induce a cytoprotective effect on ISCs. Nigro et al. artificially cultured intestinal organoids with various bacterial components commonly produced by the gut microbiota, including MDP, and observed the size and number of living organoids 4 days post-culture. It was found that the MDP-treated group has more organoids and is larger in size compared to the control group and other bacterial metabolites such as Tetra-dap, Fla., and LPS are used. It is also found that more intestinal stem cells are present in the organoids from the treated group. The MDP-treated organoid was further tested for its cytoprotective effect by observation of *in vivo* effect of doxorubicin hydrochloride, a compound toxic to intestinal stem cells, for their ability to repair the damaged intestinal epithelium in mice. Interestingly, MDP-treated mice recover much faster after 24 and 72 h compared to the MDP control and non-treated mice groups (113). Such studies have demonstrated that the bacterial metabolites produced by gut microbiota are important key players that can work both ways with the ISCS in maintaining the homeostasis of the healthy human host intestinal epithelium.

## 4. Clinical trial

Inquiries regarding relevant clinical trials are conducted in the clinicaltrials.gov database, and there are more than 150 clinical trials that are related to the either the topics of stem cells, gut microbiota or both. Upon various filtrations, currently there is only one completed clinical trial that specifically argues regarding ISC and gut microbiota. The clinical trial is mainly to investigate the relationship between ISCs and diseases related to gastrointestinal tracts. The study by Helse Fonna focuses on investigating the effects of symptoms, quality of life, fatigue, change in intestinal stem cells, enteroendocrine cells, immune system, and dysbiosis before and after fecal microbiota transplantation (FMT). FMT refers to the transfer of the intestinal microbiota of the fecal material of a healthy individual suspended in liquid form to the gastrointestinal tract of another person (114). It is the most widely used therapeutic procedure for people who suffer from an infection of the *Clostridium difficile* bacteria, which may be present when there is an imbalance in the normal gut microbiota due to repeated antibiotic treatment (114, 115). Although the exact procedures may differ from various medical institutions, the general principle remains the same in all FMT (116). Other clinical trials are correlated with FMT, but these clinical trials do not investigate the effect or relation with ISC, therefore, these clinical trials are excluded in this manuscript. Additionally, certain clinical trials are focused on the usage of other types of stem cells such as hematopoietic and peripheral blood stem cells, which are not relevant and briefly listed in Table 4.

**Table 4:** Completed and ongoing clinical trials involving FMT and different types of stem cells.

Intervention	ClinicalTrials.gov ID	Title	Objectives	Status	Duration
Intestinal stem cells	NCT03822299 (Norway)	Effects of Fecal microbiota transplantation in patients with IBS	To investigate the effects of symptoms, quality of life, fatigue, change in intestinal stem cells, enteroendocrine cells, immune system, dysbiosis before and after fecal microbiota transplantation (FMT)	Completed	01 January 2018–05 May 2019
Hematopoetic stem cell	NCT03819803 (Austria)	Fecal microbiota transplantation in aGvHD After ASCT	To investigate fecal microbiota transplantation in patients with acute gastrointestinal Graft-versus-host-disease after allogeneic hematopoetic stem cell transplantation (ASCT).	Ongoing– Phase 3	2017-03-01–Present
	NCT04593368 (Russia)	Fecal Microbiome Transplantation (FMT) in Pediatric Patients Colonized With Antibiotic-resistant Pathogens Before Hematopoietic Stem Cell Transplantation (HSCT) (FMT-HSCT)	To prospectively assess the safety and effectiveness of fecal microbiota transplantation (FMT) prior to allogeneic hematopoietic stem-cell translation (HSCT) in patients contaminated with antibiotic-resistant pathogens (ARP)	Ongoing - Phase 2	2020-12-15–Present
	NCT04269850 (Russia)	Fecal microbiota transplantation with Ruxolitinib and steroids as an upfront treatment of severe acute intestinal GVHD (JAK-FMT)	Pilot study of fecal microbiota transplantation in combination with Ruxolitinib and steroids for severe acute intestinal graft-versus-host-disease after allogeneic hematopoietic stem cell transplantation.	Ongoing– Phase 1	2019-09-01–Present
	NCT04935684 (France)	Fecal Microbiota transplantation after allogeneic stem cell transplantation (TMF-Allo)	To assess the fecal microbiota transplantation (FMT) efficacy in the prevention of allogeneic hematopoietic stem cell transplantation (allo-HSCT) complications and particularly Graft versus Host Disease (GvHD).	Ongoing– Phase 2	2022-12-20–Present
	NCT02733744 (United States)	Fecal microbiota transplantation after HSCT	To determine the feasibility of fecal microbiota transplantation (FMT) in hematopoietic stem cell transplantation (HSCT) recipients	Ongoing–Early Phase 1	2016–05–Present
Peripheral blood	NCT05873348 (China)	A controlled study on the regulation of systemic inflammation by intestinal bacteria transplantation in patients with COVID-19	To explore the regulatory effect of combined capsule FMT on the levels of inflammatory factors in peripheral blood of patients with COVID-19 during treatment.	N/A	2023-01-10–2023-04-30

Briefly, the protocol of this relevant clinical trial is carried out first by collecting the stool sample from a healthy individual and then rigorously and subsequently screened for transmissible diseases, blood and genetic screenings for specific intestinal parasites, *C. difficile*, hepatitis A-B-C, syphilis, HIV, and any other diseases chosen by local protocols (117). The screened stool sample is then diluted with PBS or other saline before the solution is homogenized and filtered to remove any solid particulates. The resulting solution is then either frozen for long-term storage or applied directly into the recipients’ intestinal tracts via colonoscopy (117). The clinical trial is also conducted in a double-blind manner to prevent discrimination and the participants were also randomly assigned into three distinct interventional groups with a placebo group (patients receiving their own fecal matter), 30 g of healthy donor fecal matter group, and 60 g of healthy donor feces. The outcome is then assessed by having the participants to answer certain questionnaires regarding their global improvement in irritable bowel syndrome symptoms, fatigue, and quality of life, all within the time frame of 3 months. However, no results have been published for this clinical trial.

## 5. Conclusions and future perspectives

The gastrointestinal tract has gone through countless epithelial turnovers and regeneration in its lifetime, but more substantial research regarding the exact mechanisms by which the proliferation and differentiation of ISCs and gut microbiota with its metabolites are interacting with each other indirectly remain to be done. The critical roles of both ISCs and gut microbiota are to maintain the normal functioning of the gastrointestinal tract, and without these two important players, the gastrointestinal tract alone would not be able to perform its crucial role in the absorption of nutrients.

**Figure 4:**
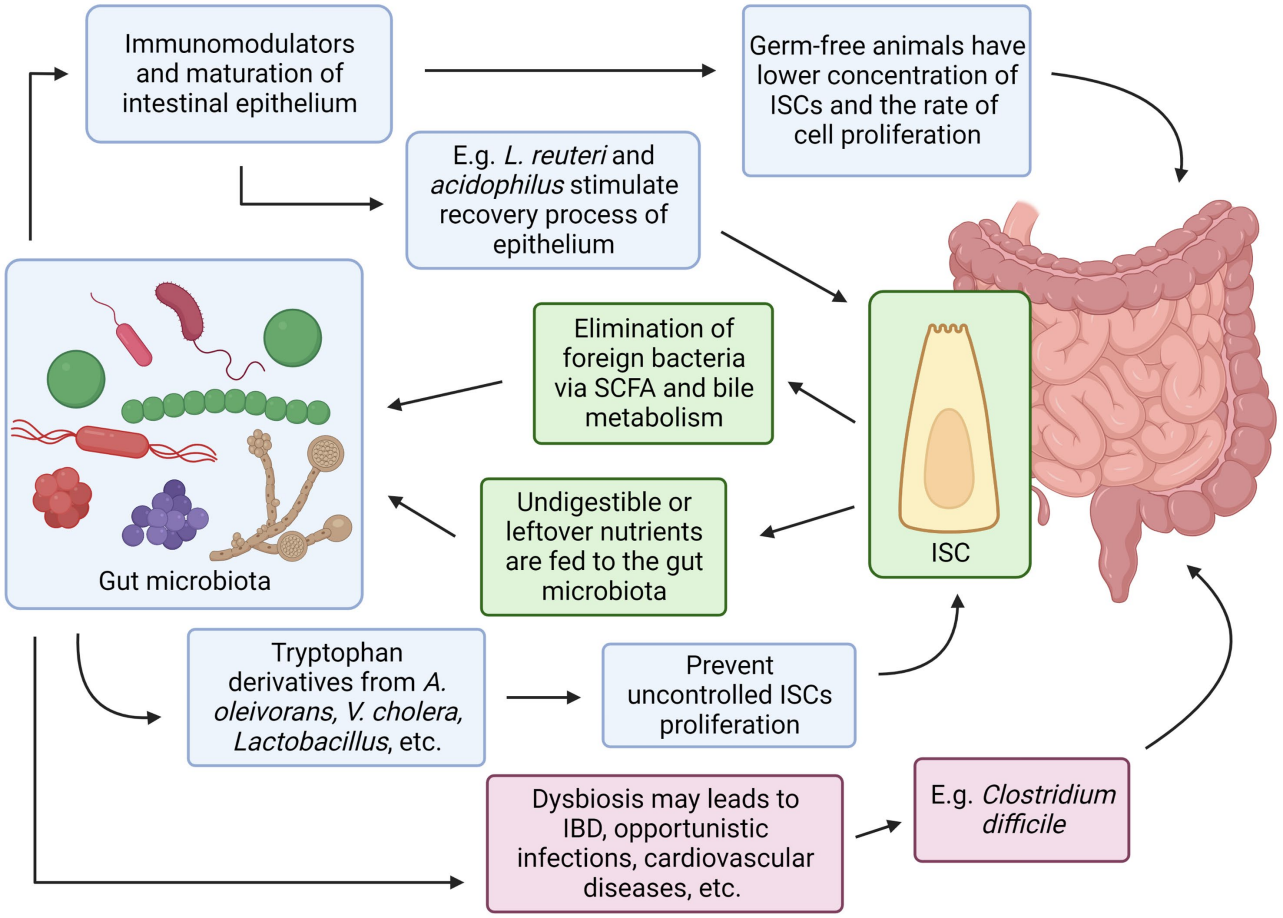
Relationships between Intestinal Stem Cells and Gut Microbiota. Created with BioRender.com. The figure is adapted with modification from Haoming Luo et al. (9).

Both intestinal stem cells and the gut microbiota have their own set of mechanisms that work in complement to each other illustrated in Figure 3. Evidence has shown some of the mechanisms were agreed upon by multiple researchers, for example, bacteria in the GI tract may provide essential substances such as SCFA that can act as additional energy sources for intestinal epithelial cells, and in response, ISC can differentiate into Paneth cells that secrete antimicrobial chemical substances and M cells that continuously sample any microbes in the GI tract, both serve to protect the indigenous gut microbiota from being eliminated by foreign microorganisms. Conversely, the absence or imbalance in the normal gut microbiota known as dysbiosis has been observed to have many derogatory effects in the host such as *C. difficile* infection that prevents the recovery and regeneration process of the intestinal epithelial, irregular hyperplastic intestinal crypts to be observed in the GI tract without essential tryptophan metabolizing bacteria to name a few (Figure 4).

Due to the microscopic size of cells and bacteria, as well as the hundreds of microbes; it is a daunting challenge to pin down every single relationship and connection of the ISCs and gut microbiota in the proper functioning of the gastrointestinal tracts, particularly of the intestines. Much has yet to be discovered. Several studies mentioned in this review involved laboratory animals or intestinal biopsies to explore ISC, gut microbiota, or both at once by inducing experimental variables in the test subjects, and subsequent results are observed. Variables that could affect the research outcome are diet diversity, the number of processed foods, and newly approved food additives in the daily diet, all of which can cause dynamic changes in an individual’s gut microbiota.

In the past, culturing bacteria commonly found in the human gastrointestinal tract has been a great challenge for biomedical scientists to taxonomically collect and classify the human gut microbiota (118, 119). Some of the bacterial species are very minuscule in number or regarded as unculturable, which can be attributed to the extreme differences in the microenvironment of inside and outside of the human gastrointestinal tract. A study by Ito et al. has successfully cultured a part of this otherwise ‘unculturable’ bacteria population using specific culture media such as chocolate agar, DHL agar, and gut microbiota medium, among others (120). However, there are still many intestinal bacteria with unknown functions that cannot be identified with conventional culturing methods.

The current technology of the next-generation sequencing (NGS) allows the identification of the genus and species of certain bacteria without depending on the lab culturing. At present, the commonly adapted NGS methodologies for sequencing bacterial DNA or RNA are shotgun metagenomic sequencing and amplicon sequencing (121). The shotgun metagenomic sequencing method uses the attachment of adapters and barcodes to fragmented bacterial DNA segments in a randomized manner. The added DNA segments are then cross-referenced with reference databases such as Genbank and Reference Sequence. Amplicon sequencing, also known as 16 s rRNA sequencing method instead amplifies a particular segment of the DNA and determines the nucleic acid sequence of the amplified product. This is always done on the 16S ribosomal RNA gene for the highly preserved sequence among bacteria and having nine hypervariable regions that are unique to each bacterial genus, thus allowing identification of the said unculturable bacteria (121).

The NGS method offers many benefits when it is applied in the clinical microbiology field. This includes a reduction in the turnaround time for the release of patient test results by the attending physicians, a wide selection of identifiable bacterial or fungal etiological agents, and less hands-on involvement of laboratory technologists, especially with slow-growing or fastidious bacteria (122). Rapid and accurate diagnosis is essential in treating patients. Approximately 60% of acute encephalitis cases have been undiagnosed, possibly due to the lack of specific assays that can detect more than 100 etiological agents of encephalitis (123). The same can be applied to other pathogenic organisms, where the NGS method can rapidly identify the presence of these organisms as opposed to individual assay kits to detect them (124). Therefore, the use of the NGS method can offer a great advantage in discovering novel microbes whose unknown or additional roles in the gastrointestinal tract can be discovered and investigated for their cellular activities.

Additionally, the gut microbiota has many essential roles in the maintenance of a properly functioning gastrointestinal tract, particularly in its anti-cancer properties. As demonstrated in chapters 2 and 3, there are many intertwining feedback systems and regulatory pathways with both the ISC and gut microbiota on the homeostasis of the intestinal epithelium and the absence of one can affect the other, which may finally result in disorganized intestinal crypts and reduced proliferation and turnover rate of the epithelium, thus impeding the intestines of its function. This leads to an interesting connection with another part of the body where there is also an indigenous microbial flora that resides outside the cells, and without this microbial community, it can subsequently affect the host and also its dependencies. An example of such a site is the human breast and its indigenous microbiota (125). Nevertheless, more studies are warranted to explore more interactions between ISC and gut microbiota, and the techniques that have been used when exploring their intricate relationships can be applied when novel studies are done on other bodily sites.

## Author contributions

FN, GT, WW, and Y-FT: conceptualization and visualization and supervision. GT, WW, and Y-FT: validation. ANAS: writing-original draft preparation. ANAS and ASJ: drawing of figures. GT, WW, FN, Y-FT, and ASJ: writing-review and editing. FN: project administration and funding acquisition. All authors have read and agreed to the published version of the manuscript.

## Funding

This review is prepared as part of the collaborative work funded by the Ministry of Higher Education Malaysia under the Fundamental Research Grant Scheme (FRGS) [Project Grant Code: DP KPT FRGS/1/2021/SKK06/UM/02/4] and a University Research Grant [FF-2023-189] from UKM.

## Conflict of interest

The authors declare that the research was conducted in the absence of any commercial or financial relationships that could be construed as a potential conflict of interest.

## Publisher’s note

All claims expressed in this article are solely those of the authors and do not necessarily represent those of their affiliated organizations, or those of the publisher, the editors and the reviewers. Any product that may be evaluated in this article, or claim that may be made by its manufacturer, is not guaranteed or endorsed by the publisher.
